# An upstream sequence modulates phenazine production at the level of transcription and translation in the biological control strain *Pseudomonas chlororaphis* 30-84

**DOI:** 10.1371/journal.pone.0193063

**Published:** 2018-02-16

**Authors:** Jun Myoung Yu, Dongping Wang, Tessa R. Ries, Leland S. Pierson, Elizabeth A. Pierson

**Affiliations:** 1 Department of Horticultural Sciences, Texas A&M University, College Station, TX, United States of America; 2 Department of Plant Pathology and Microbiology, Texas A&M University, College Station, TX, United States of America; Universita degli Studi Roma Tre, ITALY

## Abstract

Phenazines are bacterial secondary metabolites and play important roles in the antagonistic activity of the biological control strain *P*. *chlororaphis* 30–84 against take-all disease of wheat. The expression of the *P*. *chlororaphis* 30–84 phenazine biosynthetic operon (*phzXYFABCD*) is dependent on the PhzR/PhzI quorum sensing system located immediately upstream of the biosynthetic operon as well as other regulatory systems including Gac/Rsm. Bioinformatic analysis of the sequence between the divergently oriented *phzR* and *phzX* promoters identified features within the 5’-untranslated region (5’-UTR) of *phzX* that are conserved only among 2OHPCA producing *Pseudomonas*. The conserved sequence features are potentially capable of producing secondary structures that negatively modulate one or both promoters. Transcriptional and translational fusion assays revealed that deletion of 90-bp of sequence at the 5’-UTR of *phzX* led to up to 4-fold greater expression of the reporters with the deletion compared to the controls, which indicated this sequence negatively modulates phenazine gene expression both transcriptionally and translationally. This 90-bp sequence was deleted from the *P*. *chlororaphis* 30–84 chromosome, resulting in 30-84Enh, which produces significantly more phenazine than the wild-type while retaining quorum sensing control. The transcriptional expression of *phzR/phzI* and amount of AHL signal produced by 30-84Enh also were significantly greater than for the wild-type, suggesting this 90-bp sequence also negatively affects expression of the quorum sensing genes. In addition, deletion of the 90-bp partially relieved RsmE-mediated translational repression, indicating a role for Gac/RsmE interaction. Compared to the wild-type, enhanced phenazine production by 30-84Enh resulted in improvement in fungal inhibition, biofilm formation, extracellular DNA release and suppression of take-all disease of wheat in soil without negative consequences on growth or rhizosphere persistence. This work provides greater insight into the regulation of phenazine biosynthesis with potential applications for improved biological control.

## Introduction

Phenazines are bacterial secondary metabolites produced by a diversity of plant-associated *Pseudomonas* species and that contribute to their ability to promote plant health [1–8]. *Pseudomonas chlororaphis* 30–84 is a rhizosphere-colonizing bacterial species capable of producing an array of secondary metabolites with beneficial agronomic applications. *P*. *chlororaphis* 30–84 was isolated as a biological control agent for take-all disease of wheat caused by the fungal pathogen *Gaeumannomyces graminis* var. *tritici* (*Ggt*). Phenazines are the principal antifungal secondary metabolites produced by *P*. *chlororaphis* 30–84 and several other well-studied biological control agents [1–7]. Phenazines are redox active molecules that are involved in diverse biological functions [8, 9]. *P*. *chlororaphis* 30–84 produces three phenazine derivatives, phenazine-1-carboxylic acid (PCA), 2-hydroxy-phenazine-1-carboxylic acid (2OHPCA) and a small amount of 2-hydroxy-phenazine (2OHPZ). PCA is produced via expression of the phenazine biosynthetic operon *phzXYFABCD*, and the phenazine modifying gene *phzO*, encoding an aromatic monooxygenase, is responsible for the conversion of PCA to the 2-hydroxy derivatives [1, 8, 10].

The ecological benefits of phenazine production have been well documented (reviewed in [8, 9, 11, 12]). These include their antibiotic characteristics, which facilitate survival in competition with other microorganisms [8, 9, 13, 14]. Phenazine production by *P*. *chlororaphis* 30–84 is primarily responsible for the inhibition of *Ggt in vitro* and *in situ* on roots as well as for its persistence on roots in competition with other rhizosphere microorganisms [1, 13]. Phenazines also contribute to biofilm formation [15–19]. In *P*. *chlororaphis* 30–84 this was demonstrated using a phenazine biosynthetic mutant of *P*. *chlororaphis* 30–84, 30-84ZN (*phzB*::*lacZ*), which was defective in cell attachment and biofilm development [17]. Subsequently, using isogenic derivatives of *P*. *chlororaphis* 30–84 producing only PCA or overproducing 2OHPCA, the roles of different phenazines in specific aspects of biofilm formation and architecture were demonstrated [16]. More recently, differences in the ecological roles and transcriptional influence of each phenazine derivative produced by *P*. *chlororaphis* 30–84 were reported [19], including that 2OHPCA production more readily promotes extracellular DNA release, which results in a greater structured biofilm matrix. RNA-seq analysis revealed that phenazine production has broad impacts on gene expression patterns, including genes involved in the biosynthesis of exoenzymes, secondary metabolites, and other genes important for survival. Similar results have been shown for other phenazine producing species [20].

In most phenazine-producing bacteria including the plant growth promoting rhizosphere colonists *P*. *synxantha* (formerly *P*. *fluorescens*) 2–79, *P*. *chlororaphis* PCL1391 and *P*. *chlororaphis* 30–84, phenazine biosynthesis is controlled directly by the PhzR/PhzI quorum sensing system [21–25]. Typically, *phzR* and *phzI* are located immediately upstream of the phenazine biosynthetic operon. The gene *phzI* encodes an *N-*acyl homoserine lactone (AHL) synthase and *phzR* encodes a transcriptional regulator of the *phz* biosynthetic operon [22, 24, 25]. Once AHL signals reach a threshold level, they interact with PhzR forming an active complex that binds to the specific sequence motif known as a “*phz* box” within the phenazine biosynthetic promoter resulting in the activation of the expression of the phenazine biosynthetic genes. The activated PhzR-AHL complex also binds to a *phz* box in the promoter region of *phzI* to enhance *phzI* expression resulting in increased AHL signal production.

The GacS/GacA two-component global regulatory system (TCS) is essential for biosynthesis of bacterial secondary metabolites including phenazines, AHL signals, exoprotease, lipase, gelatinase and HCN in *P*. *chlororaphis* 30–84 [26, 27] and other *Pseudomonas* species [28–30]. In *Pseudomonas*, the Gac/Rsm system is comprised of three small non-coding RNAs (ncRNA, e.g. rsmX, rsmY and rsmZ) and two RNA binding repressor proteins (RsmA and RsmE) [27, 28, 31–33]. The RsmA/E proteins function as posttranscriptional repressors by binding to a specific sequence motif (e.g. -GGA- or ribosome binding site) in the mRNA and blocking translation initiation and/or targeting mRNA degradation [32, 34, 35]. The binding of the ncRNAs to RsmA and RsmE results in sequestration of these repressor proteins and alleviation of their translational regulation [27, 36–38]. For example in *P*. *chlororaphis* 30–84, the Gac system controls the expression of *rsmZ*, and in turns activates the expression of *phz* genes by titrating the translational repressor RsmE [27]. In addition to quorum sensing and the Gac/Rsm network, other regulatory genes are involved in the regulation of phenazine biosynthesis in *P*. *chlororaphis* 30–84 including, sigma factor RpoS, the two component system RpeA/RpeB, and the transcription regulator Pip [26, 27, 39, 40].

**Fig 1:**
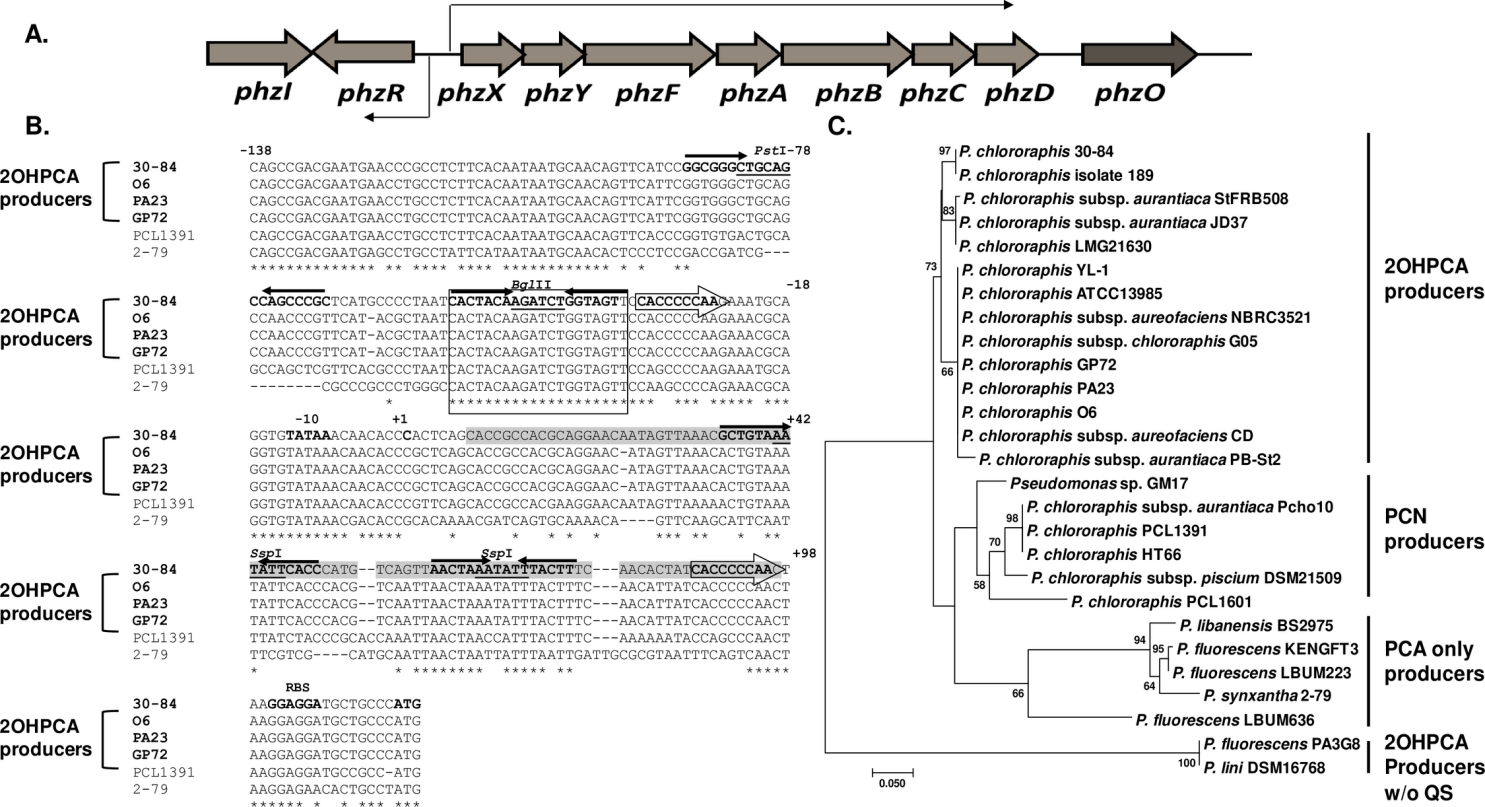
Comparison of the nucleotide sequences of the phenazine biosynthetic promoter regions. **(A)** Quorum sensing genes *phzR* and *phzI* are located immediately upstream of the phenazine biosynthetic operon and arrows indicate divergent transcription of *phzR* and *phzX* (**B)** The boxed region indicates the putative *phz* box sequences for the phenazine biosynthetic promoter of the six different phenazine-producing strains. Restriction enzyme sites are underlined. The putative -10 sequences, transcription start site (+1), ribosome binding site sequences (RBS), and ATG of PhzX are bolded. The hollow arrows indicate the direct repeat sequences (CACCCCCAA). Solid arrows indicate the four palindromic sequences. The 90-bp of 5’-UTR of phenazine biosynthetic operon is grey highlighted. The asterisks (*) indicate fully conserved residues, and gaps introduced for alignment are indicated by dashes (-). DNA sequences were obtained from National Center for Biotechnology Information (NCBI) (https://www.ncbi.nlm.nih.gov/) and aligned using Clustal Omega (http://www.ebi.ac.uk/Tools/msa/clustalo/). (**C)** Maximum-Likelihood (ML) tree based on a 250 bp region upstream from the translation start site of the phenazine biosynthetic operon from 27 different phenazine-producing pseudomonads. Sequences were retrieved from the *Pseudomonas* Genome database (www.pseudomonas.com) and NCBI. The tree with the highest log likelihood (-1149.3348) is shown, and only ML bootstrap values ≥ 50% are shown at nodes.

Predictably, much attention related to the regulation of phenazine biosynthesis has focused on the integration of these additional circuits as a regulatory network with quorum sensing to control phenazine production. In the present study, bioinformatics analysis was used to compare the 430-bp intergenic region between the divergently oriented *phzR* and *phzX* promoters in *P*. *chlororaphis* 30–84 (Fig 1A) and other closely related phenazine producing strains. This region includes the previously defined 18-bp palindromic *phz* box (and -10 hexamer sequence), which is highly conserved with previously identified *phz* box sequences in the phenazine operon promoters of biological control agents *P*. *synxantha* 2–79 and *P*. *chlororaphis* PCL1391 [22, 23, 41]. The *P*. *chlororaphis* 30–84 sequence motifs located within the 5’-UTR between the *phz* box and the ATG start codon of the first gene of the phenazine operon (*phzX*) differ from the two strains that do not produce 2OHPCA, *P*. *synxantha* 2–79 and *P*. *chlororaphis* PCL1391, but are similar to other strains that do produce 2OHPCA. These sequences are predicted to generate significant secondary structure. We examined the role of this region in phenazine regulation by creating a series of transcriptional and translation fusion plasmid derivatives with deletions or alterations aimed at disrupting the predicted secondary structures. We discovered a sequence within the 5’-UTR of *P*. *chlororaphis* 30–84, which is conserved among 2OHPCA producers, that modulates phenazine gene expression via transcriptional and translational regulation. The effects of the deletion of this sequence on *P*. *chlororaphis* 30–84 phenazine gene expression and on biological control efficacy were investigated.

## Results

### Analysis of the sequence between *phzR* and the phenazine biosynthetic operon in *P*. *chlororaphis* 30–84 and comparison to other phenazine producing strains

Sequence analysis of the promoter region and 5’-UTR of the phenazine biosynthetic operon in *P*. *chlororaphis* 30–84 revealed that there are four palindromic sequences: one centered on the *Pst*I site (5’-GCGGGCTGCAGCCAGCCCGC-3’, Fig 1B: -87 to -67), a second centered on the *Bgl*II site (5’-CACTACAAGATCTGGTAGT-3’, Fig 1B: -54 to -35), a third on the first *Ssp*I site (5’-GCTGTAAATATTCACC-3’, Fig 1B: +35 to +52), and the fourth centered on the second *Ssp*I site (5’-AACTAAATATTTACTT-3’, Fig 1B: +61 to +77). The sequence surrounding the *Bgl*II site is a perfect match with the *phz* box found in other phenazine producing *Pseudomonas* strains that contain a single phenazine biosynthetic operon in their genome [23, 42]. The -10 site is located 9 bp upstream of transcription start site (TSS), which was previously identified by RNA-seq analysis [27] with a good sequence match to consensus sequences. Located between the *phz* box and the translation start site (ATG) of *phzX* are two identical repeats (5’-CACCCCCAA-3’, Fig 1B: -33 to -24 and +87 to +96) flanking a 112 bp sequence that has 38.4% GC content (as compared to the 51% GC content throughout the rest of the region and 62.9% GC overall content in the *P*. *chlororaphis* 30–84 genome) [43].

We compared sequence in this region (Fig 1B: -138 to the ATG) to sequence in the same region in other phenazine producing strains that have a single *phz* operon in their genome including *P*. *chlororaphis* O6, PA23 and GP72 (producing PCA, 2OHPCA and 2OHPZ), *P*. *chlororaphis* PCL1391 [producing PCA and phenazine-1-carboxamide (PCN)] and *P*. *synxantha* 2–79 (producing PCA only). The sequence surrounding the promoter region is highly conserved in all six strains, e.g., the sequence of the *phz* box and -35 and -10 regions match perfectly (Fig 1B). However, the palindromic sequence surrounding the *Pst*I site is highly conserved only in the other 2OHPCA producing strains *P*. *chlororaphis* O6, PA23 and GP72, is less conserved in *P*. *chlororaphis* PCL1391 and is absent in *P*. *synxantha* 2–79 (Fig 1B). The 5’-UTR also is highly conserved only among the 2OHPCA producing strains with similar GC content (38.7% GC in *P chlororaphis* O6, PA23 and GP72) and all contain the direct repeats and repetitive sequence motifs (*Ssp*I sites) (Fig 1B). Although a similar region with low GC content is present in the 5’-UTR of *phzA* in *P*. *synxantha* 2–79 (40% GC) and *P*. *chlororaphis* PCL1391 (38% GC), both strains lack these direct repeats. The third palindromic sequence surrounding the first *Ssp*I site is identical to the sequence of 2OHPCA producing strains *P*. *chlororaphis* O6, PA23 and GP72, but is less conserved in *P*. *synxantha* 2–79 and *P*. *chlororaphis* PCL1391. The last palindromic sequence surrounding the second *Ssp*I site is highly conserved even though the *Ssp*I site is not present in *P*. *synxantha* 2–79 or *P*. *chlororaphis* PCL1391.

To examine whether similarities in this region are related to the types of phenazines each strain produces, we expanded the comparison to include twenty seven other phenazine producing pseudomonads that have a single phenazine operon in their chromosome. Based on Maximum Likelihood analysis, all the phylograms sorted into 4 major groups (Fig 1C). The 2OHPCA producing strains (with *phzO* located downstream of the *phz* operon) including *P*. *chlororaphis* 30–84, O6, PA23 and GP72 grouped together and formed a 2OHPCA producer clade with moderate bootstrap support. The second clade contained PCN producing strains (with *phzH* located downstream of the *phz* operon) including *P*. *chlororaphis* PCL1391, and PCA only producing strains (lacking any phenazine modifying gene) including *P*. *synxantha* 2–79 grouped together and formed a PCA producer clade with moderate bootstrap support. The forth clade included the two 2OHPCA producing strains without the quorum sensing system upstream of *phz* operon.

### Role of conserved sequence motifs on phenazine gene expression in *P*. *chlororaphis* 30–84

**Fig 2:**
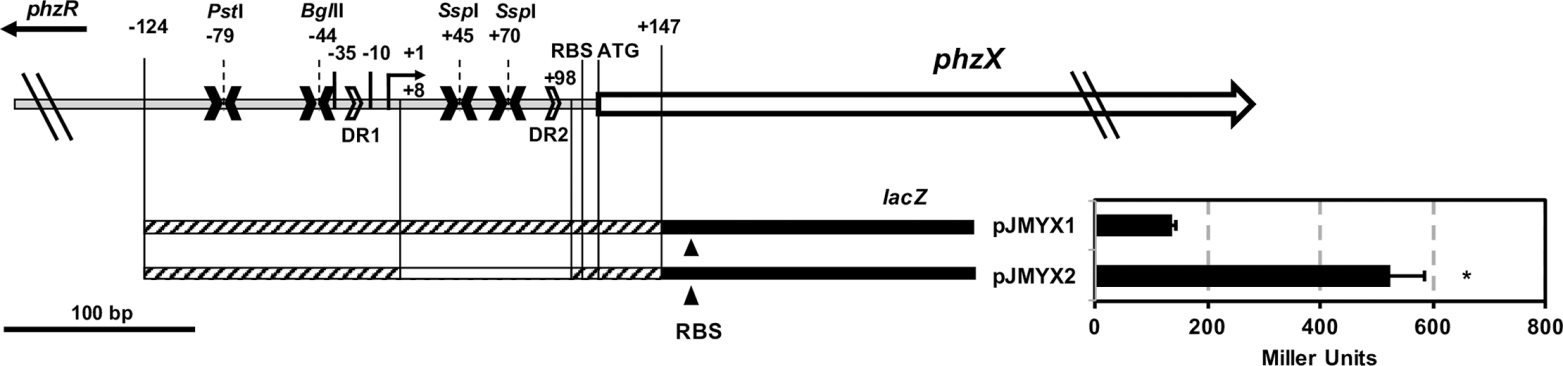
Effect of 90-bp deletion of the 5’-UTR in *phzX* expression using a transcriptional fusion to the *lacZ* reporter gene. The region of the *P. chlororaphis* 30–84 chromosome that contains the promoter of the phenazine biosynthetic operon (*phzXYFABCD*), four palindromic sequences (solid arrowheads) and two direct repeats (open arrowheads). Construction map for transcriptional fusion reporter plasmids pJMYX1 (control) and pJMYX2 (90-bp deletion): the rectangles with the diagonal lines indicate the sequence included in each derivative, the hollow rectangles indicate the 90-bp region not included in pJMYX2, the solid black rectangle represents the *lacZ* reporter gene sequence, and the black triangles under the *lacZ* reporter represent the ribosome binding site of *lacZ*. The transcriptional expressions of each reporter plasmids in 30-84Ice were determined via the β-galactosidase activities and presented as Miller Units. Data represent the average of eight replicates with standard errors. Asterisks indicate significant differences as determined by unpaired t-test (P < 0.05).

To investigate the potential role of the sequence motifs in *P*. *chlororaphis* 30–84 on phenazine gene expression, a series of transcriptional fusion plasmid derivatives with deletions in the different features were cloned into the promoter trap vectors (pGT2-*lacZ* or pKT2-*lacZ*), resulting in the plasmids pJMYX1 (control) and pJMYX2 (containing a deletion of 90-bp in the 5’-UTR of phenazine biosynthetic operon) (Table 1 and Fig 2) and pJMYX3 (control), pJMYX4 (disruption of the first palindrome) and pJMYX5 (disruption of *phz* box) (Table 1 and S1 Fig). Each plasmid was introduced separately into 30-84Ice (*phzB*::*inaZ*), a phenazine non-producing mutant employed so that phenazine production did not interfere with the β*-*galactosidase assays.

**Table 1:** Bacterial strains and plasmids used in this study.

Strains and plasmids	Relevant characteristics	Reference or source
***Pseudomonas chlororaphis***		
30-84WT	Phz^+^ Rif^R^ wild-type	[1]
30-84ZN	Phz^-^ Rif^R^ *phzB*::*lacZ* genomic fusion	[44]
30-84Ice	Phz^-^ Rif^R^ *phzB*::*inaZ* genomic fusion	[44]
30-84Enh	Phz^+^ Rif^R^ 90-bp deletion at the 5’-UTR of *phzX*	This study
30-84ZN-Enh	Phz^-^ Rif^R^ *phzB*::*lacZ* genomic fusion and 90-bp deletion at the 5’-UTR of *phzX*	This study
30-84W	Phz^-^ Rif^R^ spontaneous *gacA* mutant	[26]
30-84I/Z	Phz^-^ Rif^R^ *phzB*::*lacZ* and *phzI*::Km^R^	[39]
30-84RsmE	Phz^+^ Rif^R^ Km^R^ *rsmE*::Km^R^	This study
***E*. *coli***		
DH5α	F^-^ *recA1 endA1 hsdR17 supE44 thi-1 gyrA96 relA1* Δ(*argF-lacZYA*) *I169* Φ80*lacZ* ΔM15λ^-^	GIBCO-BRL
HB101	F^-^ *hsdS20*(r_B_^-^ m_B_^-^) *supE44 recA1 ara14 proA2 lacY1 galK2 rpsL20 xyl-5 mtl-5*λ^-^	GIBCO-BRL
**Plasmids**		
pKT2*lacZ*	Km^R^ pVS1-p15A shuttle vector for constructing the transcriptional *lacZ* fusions, pPROBE-KT'	[40]
pGT2*lacZ*	Gm^R^ pVS1-p15A shuttle vector for constructing the transcriptional *lacZ* fusions, pPROBE-GT'	[45]
pJMYX1	Transcriptional *phzX-lacZ* fusion containing a 271 bp fragment (-124 to +147) in pGT2*lacZ*	This study
pJMYX2	Transcriptional *phzX-lacZ* fusion containing a 181 bp fragment (-124 to +147) with a deletion of 90-bp (+8 to +98) in pGT2*lacZ*	This study
pJMYX3	Transcriptional *phzX-lacZ* fusion containing a 354 bp fragment (-124 to +230) in pKT2*lacZ*	This study
pJMYX4	Transcriptional *phzX-lacZ* fusion containing a 310 bp fragment (-79 to +230) in pKT2*lacZ*	This study
pJMYX5	Transcriptional *phzX-lacZ* fusion containing a 274 bp fragment (-44 to +230) in pKT2*lacZ*	This study
pJMYX2-DR2	Site mutagenesis of the second direct repeat of *phzX* promoter region on pJMYX4	This study
pJMYX2-DR12	Site mutagenesis of the both direct repeat of *phzX* promoter region on pJMYX4	This study
pME6015	Tc^R^ pVS1-p15A shuttle vector for constructing the translational *lacZ* fusions	[34]
pJMYX6	Translational *phzX-lacZ* fusion containing a 299 bp fragment (-124 to +175) in pME6015	This study
pJMYX7	Translational *phzX-lacZ* fusion containing a 209 bp fragment (-124 to +175) with deletion of 90-bp (+8 to +98) in pME6015	This study
pJMYR1	Transcriptional *phzR-lacZ* fusion containing a 443 bp fragment (-297 to +134) in pGT2*lacZ*	This study
pJMYR2	Transcriptional *phzR-lacZ* fusion containing a 443 bp fragment (-297 to +134) with a deletion of 90-bp (+8 to +98) in pGT2*lacZ*	This study
pUC57-Enh	pUC57 containing a 1.5-kb fragment including middle of *phzR* to *phzY* with a deletion of 90-bp of the 5’-UTR of phenazine biosynthetic operon	GenScript
pLAFR3	IncP1 *cos*^*+*^ *rlx*^*+*^ Tc^R^	[46]
pLAF-Enh	pLAFR3 containing a 1.5-kb fragment including middle of *phzR* to *phzY* with a deletion of 90-bp of the 5’-UTR phenazine biosynthetic operon	This study
pEX18Ap	Ap^R^ Gene replacement vector with MCS from pUC18	[47]
pEX18Ap-rsmE	pEX18Ap containing a 1307 bp fragment flanking the *rsmE* gene	This study
pEX18Ap-rsmEKO	Km^R^ pEX18RsmE containing a 961 bp Km resistance cassette fragment	This study
pUC4K	Km^R^ Ap^R^ containing a kanamycin resistance cassette (*aph*)	[48]

^a^ Km^R^, Ap^R^, Gn^R^ Tc^R^ and Rif^R^ = kanamycin, ampicillin, gentamicin, tetracycline and rifampicin resistance, respectively.

The greatest increase in β*-*galactosidase activity relative to the appropriate control was observed for pJMYX2, which had 4-fold higher expression than the control plasmid pJMYX1 (Fig 1). Plasmid pJMYX2 has a 90-bp deletion, resulting in the removal of both *Ssp*I sites and the second direct repeat (+8 from the TSS to the ribosome binding site). This result indicates that the 5’-UTR of the phenazine biosynthetic operon negatively modulates phenazine expression.

The secondary structure predictive program Mfold [49] identified the second direct repeat (5’-CACCCCCAA-3’) in the 5’-UTR of phenazine biosynthetic operon as potentially capable of providing significant secondary structure in mRNA, which may contribute to the observed reduced expression (S2A Fig). To determine whether this secondary structure affected phenazine expression, plasmid derivatives were constructed with nucleotide alterations in the second direct repeat or both direct repeats (5’-CACCCCCAA-3’ to 5’-CACTATCAT-3’, S2B Fig). However, there were no significant differences in β-galactosidase expression with modifications to the direct repeats. The results indicated that small nucleotide sequence modifications were insufficient to relieve reduced expression. Predictions of potential secondary structure by Mfold were consistent with these derivatives altering but not eliminating possible secondary structures in this region.

Interestingly, β*-*galactosidase activity for pJMYX4 (missing half of the first palindromic sequence) was ~2-fold higher expression than for the control plasmid pJMYX3 (containing all palindromic sequences), suggesting that the first palindrome also may negatively affect phenazine expression to lesser extent under the conditions used (S1 Fig). Plasmid pJMYX5 (missing half of the *phz* box) had no β*-*galactosidase activity confirming that an intact *phz* box is required for phenazine promoter function (S1 Fig).

### Deletion of 90-bp of the 5’-UTR of phenazine biosynthetic operon resulted in enhanced gene expression and phenazine production

**Fig 3:**
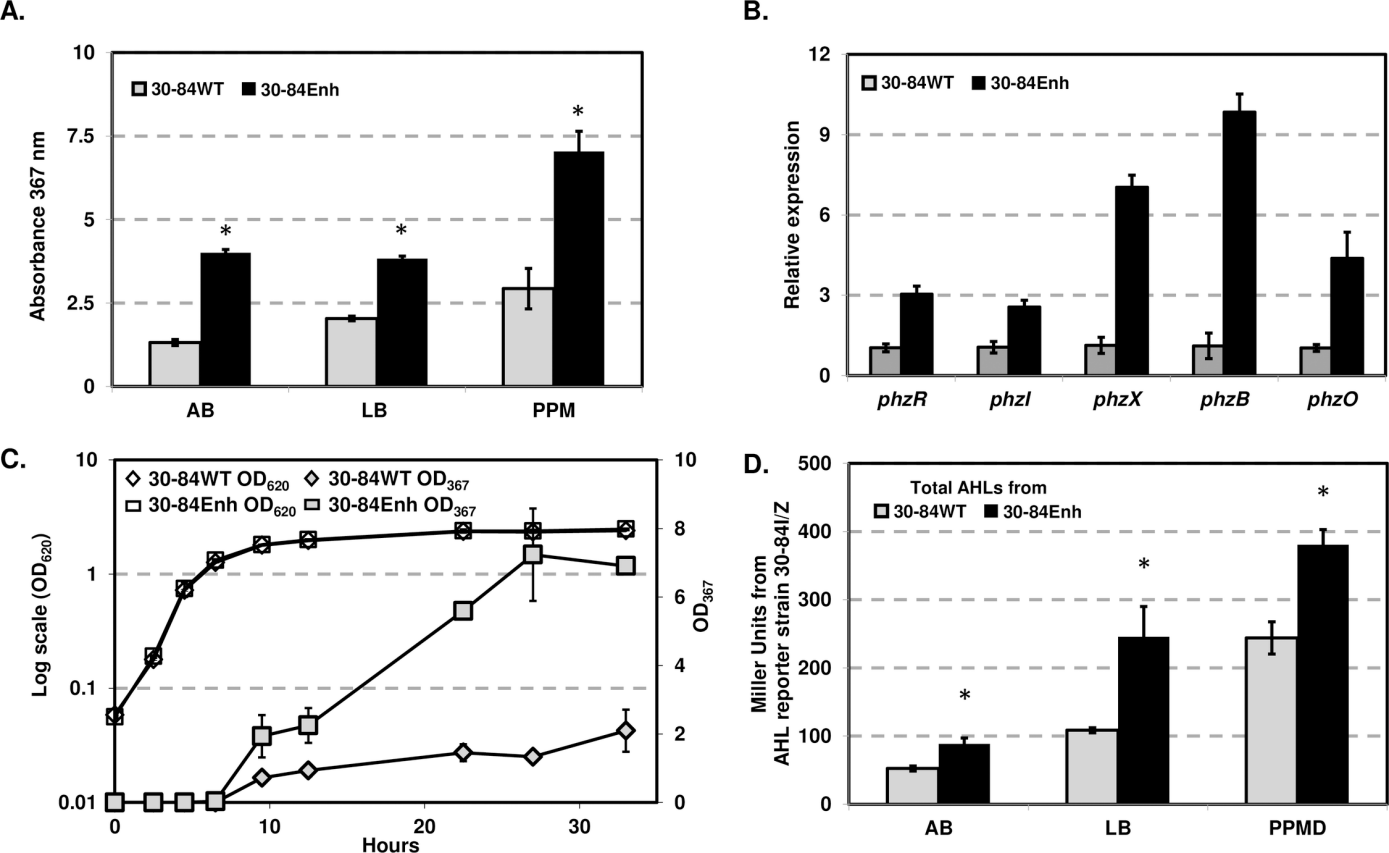
Phenazine production and gene expression patterns. **(A)** Phenazine production by the 30–84 wild-type (WT) and 30-84Enh in different media (AB minimal, LB and PPMD). Data points represent means of three replicates ± standard error. Asterisks indicate significant differences as determined by unpaired t-test (P < 0.05). Experiments were repeated twice. (**B)** Expression of the phenazine regulatory genes in 30-84WT and 30-84Enh. The relative expression of selected *phz* operon (*phzX*, *phzB* and *phzO*) in 30-84WT and 30-84Enh were determined by qPCR using the16s rDNA gene as the reference. **(C)** Time course of phenazine production by 30-84WT and 30-84Enh in AB-C medium. During the growth, samples were taken periodically and from them total phenazines were extracted. Data points represent means of three replicates ± standard error. In some cases, error bars do not exceed the size of the symbol. Experiments were repeated twice. (**D)** AHL production by 30-84WT and 30-84Enh. AHLs obtained from 30-84WT and 30-84Enh were quantified using the AHL-specific reporter strain 30-84I/Z (*phzI* ^*-*,^ *phzB*::*lacZ*). AHLs were quantified based on β-galactosidase activity and reported in Miller Units (MU). Data are the means and standard errors of 8 replicates. Asterisks indicate significant differences as determined by unpaired t-test (P < 0.05). (**A)** and (**C)** Phenazines were quantified by UV-visible light spectroscopy at absorbance of 367 nm.

Chromosomal deletions of the 90-bp sequence in the 5’-UTR of the phenazine biosynthetic operon were constructed via homologous recombination in the wild-type and the phenazine mutant 30-84ZN, resulting in derivatives 30-84Enh and 30-84ZN-Enh, respectively (Table 1). Compared to the wild-type, strain 30-84Enh produced significantly more phenazine in different types of media (i.e. 3.1-fold, 1.9-fold, and 2.4-fold in AB, LB and PPMD, respectively, Fig 3A and S3A Fig). Also β-galactosidase activity of 30-84ZN-Enh was 3.2-fold higher than that of 30-84ZN, indicating this was a result of enhanced expression of the phenazine biosynthetic genes (S3B and S3C Fig). Transcript abundances of *phzX*, *phzB*, and the phenazine modifying gene *phzO* measured via quantitative PCR (qPCR) were also greater in 30-84Enh relative to the wild-type (Fig 3B). These results suggest that the 90-bp of the 5’-UTR negatively modulates transcript abundance of the phenazine biosynthetic operon.

In order to determine whether deletion of the 90-bp of the 5’-UTR of phenazine biosynthetic operon affected quorum sensing control of phenazine gene regulation, the relationship between the cell growth and the production of phenazine was followed over time. The wild-type and 30-84Enh had similar growth rates, indicating that alteration of the 5’-UTR of phenazine biosynthetic operon and enhanced phenazine production did not alter growth rates *in vitro* (Fig 3C). Neither strain produced detectable amounts of phenazines below OD_620_ = 0.8, indicating both strains lack phenazine production at low cell density. Phenazine production by 30-84Enh was significantly greater than the wild-type after OD_620_ = 1.8, and by late stationary phase the phenazine concentration from strain 30-84Enh was 5.8-fold higher than the wild-type (Fig 3C). Importantly, the requirement for quorum sensing was further verified by introducing plasmids pJMYX1 and pJMYX2 into the AHL synthase defective mutant 30-84I. No β-galactosidase activity was detected from 30-84I harboring either plasmid. These results indicate that loss of the 90-bp region did not relieve the requirement for quorum sensing activation as evidenced by the kinetics of phenazine production and the requirement for a functional *phzI* for gene expression.

### Deletion of the 90-bp of the 5’-UTR of phenazine biosynthetic operon results in enhanced expression of quorum sensing

Given that *phzR* is located immediately upstream of *phzX* and divergently transcribed (Fig 1A), alterations in adjacent regulatory elements may influence the transcription of *phzR*, which may in turn lead to changes in the transcription of *phzI*, since *phzI* also has a *phz* box associated with its promoter region. To measure *phzR* expression, *phzR* transcriptional fusion vectors pJMYR1 (control, with the 90-bp region) and pJMYR2 (deletion of the 90-bp region) were constructed (Table 1. and S4 Fig). The β-galactosidase assays showed that *lacZ* gene expression was slightly, but statistically higher (p < 0.005, n = 8) in pJMYR2 (205 ± 4 MU) compared to the control, pJMYR1 (134 ± 4 MU). In addition, quantification of total AHL produced by the wild-type and 30-84Enh in different medium revealed that 30-84Enh produced significantly more AHL than the wild-type (Fig 3D). The transcript abundances of *phzR* and *phzI* were also greater in 30-84Enh compared to the wild-type as verified by qPCR (Fig 3B). These data indicate that the 90-bp region also has a negative influence on *phzR* expression which in turn reduces *phzI* expression, and that removal of the region enhanced the expression of the quorum sensing system.

### Interaction between Gac/RsmE system and the 5’-UTR of phenazine biosynthetic operon

**Fig 4:**
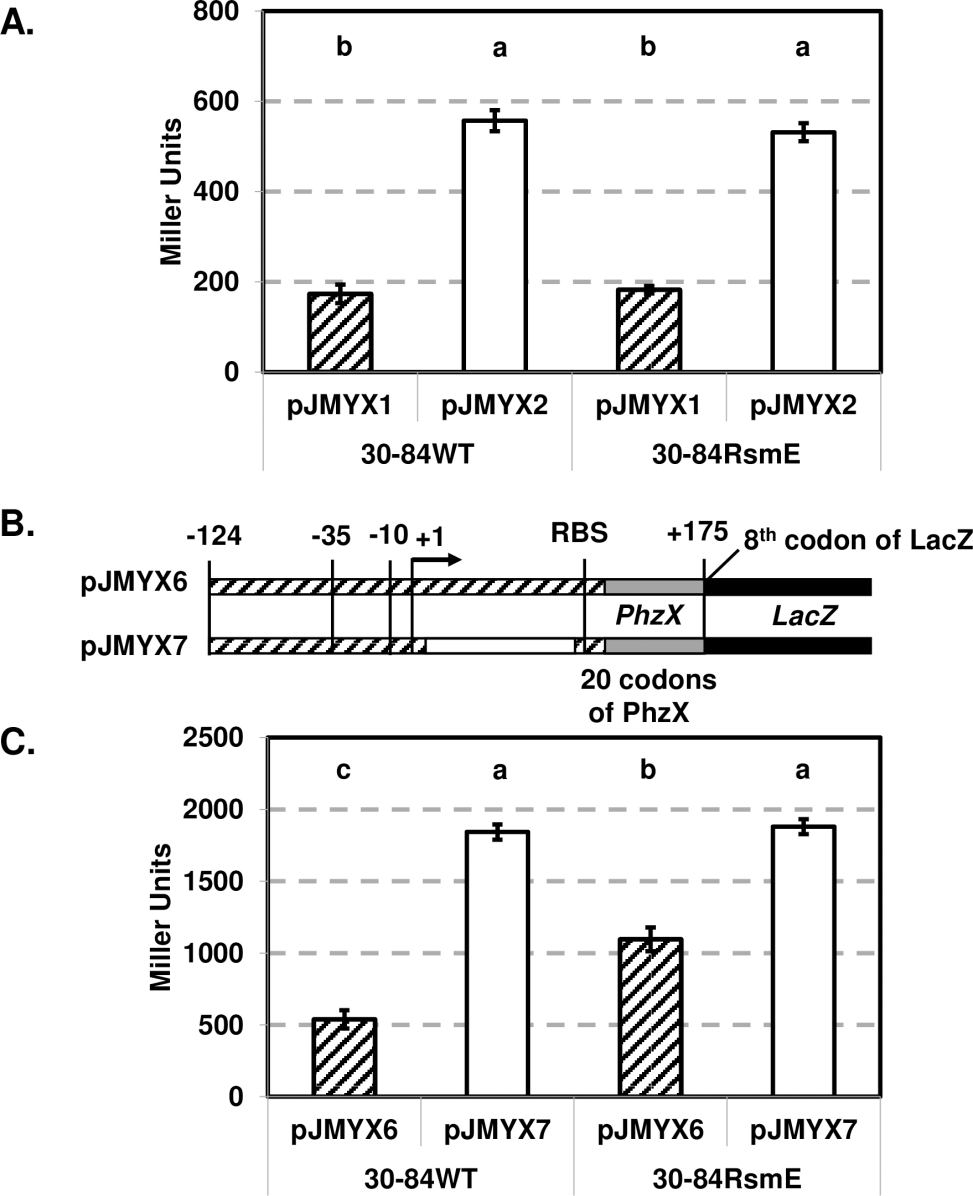
Interaction between the 5’-UTR of phenazine biosynthetic operon and post-transcriptional repressor RsmE. (**A)** The β-galactosidase activity of pJMYX1 and pJMYX2 in 30–84 wild-type (WT) and 30-84RsmE. Transcriptional activities of each reporter are expressed in Miller Units as the average of 8 replicates with standard error. Values with the same letter do not differ significantly as determined by a Fishers protected Least Significantly Difference (LSD) test (P ≥ 0.05). **(B)** Construction map for translational fusion reporter plasmids: the rectangles with the diagonal lines indicate the sequence included in each derivative, the hollow rectangle indicates the 90-bp regions not included in pJMYX7, the solid grey rectangle represents 20 codons of PhzX and the solid black rectangle represents the *lacZ* reporter gene sequence. (**C)** The β-galactosidase activity of pJMYX6 and pJMYX7 in the 30-84WT and 30-84RsmE. Translational activities of each reporter are expressed in Miller Units as the average of 8 replicates with standard error. Values with the same letter do not differ significantly as determined by a Fishers protected Least Significantly Difference (LSD) test (P ≥ 0.05).

To determine whether the Gac/RsmE system negatively influences transcriptional expression via interaction with the 5’-UTR of phenazine biosynthetic operon, plasmids pJMYX1 (control) and pJMYX2 (90-bp deletion) were introduced separately into the wild-type, 30-84W (a spontaneous *gacA* mutant) and 30-84RsmE (*rsmE*::Km^R^). The β-galactosidase activities of both plasmids in 30-84W were below the detectable amount, whereas β-galactosidase activity of pJMYX2 was higher than pJMYX1 in the wild-type (consistent with the previous experiments) and in the RsmE mutant (Fig 4A). The results suggest that pJMYX2 requires a functional Gac system to activate the *phzX* expression and also indicate that loss of *rsmE* has no effect on either pJMYX1 or pJMYX2 transcription. Since RsmE is a post-transcriptional regulator, we constructed the translational fusion reporter plasmids pJMYX6 (control) and pJMYX7 (90-bp deletion) (Table 1 and Fig 4B). Sequence spanning -124 to +175 relative to the TSS of *phzX* (includes first 20 codons of PhzX) with and without with the 90-bp sequence in the 5’-UTR of phenazine biosynthetic operon were fused in frame with the 8^th^ codon of *lacZ* to create the translational fusions pJMYX6 (control) and pJMYX7 (90-bp deletion), respectively, in pME6015 [34, 45]. The β-galactosidase activity (translational expression) of pJMYX7 (1743 ± 46 MU) was 3.2-fold higher than pJMYX6 (540 ± 63 MU) in the wild-type background (Fig 4C), which is similar to the relative fold change in transcriptional expression between pJMYX1 and pJMYX2 in the wild-type (Fig 4A). The β-galactosidase activity of pJMYX7 (90-bp deletion) was similar in both wild-type and the *rsmE* mutant backgrounds, indicating that loss of *rsmE* has no effect on pJMYX7 translation (Fig 4C). Importantly, the β-galactosidase activity of JMYX6 was significantly greater in the *rsmE* mutant compared to the wild-type, but was still significantly less than the activity of pJMYX7 in the *rsmE* mutant, indicating disruption of *rsmE* relieves, but only partially, repression of pJMYX6. Together the results using the transcriptional and translational fusions indicate that the 90-bp sequence is involved in RsmE-mediated translational regulation, but this does not explain entirely the effect of this region on phenazine expression.

### Ecological role of 90-bp deletion of the 5’-UTR of phenazine biosynthetic operon

**Fig 5:**
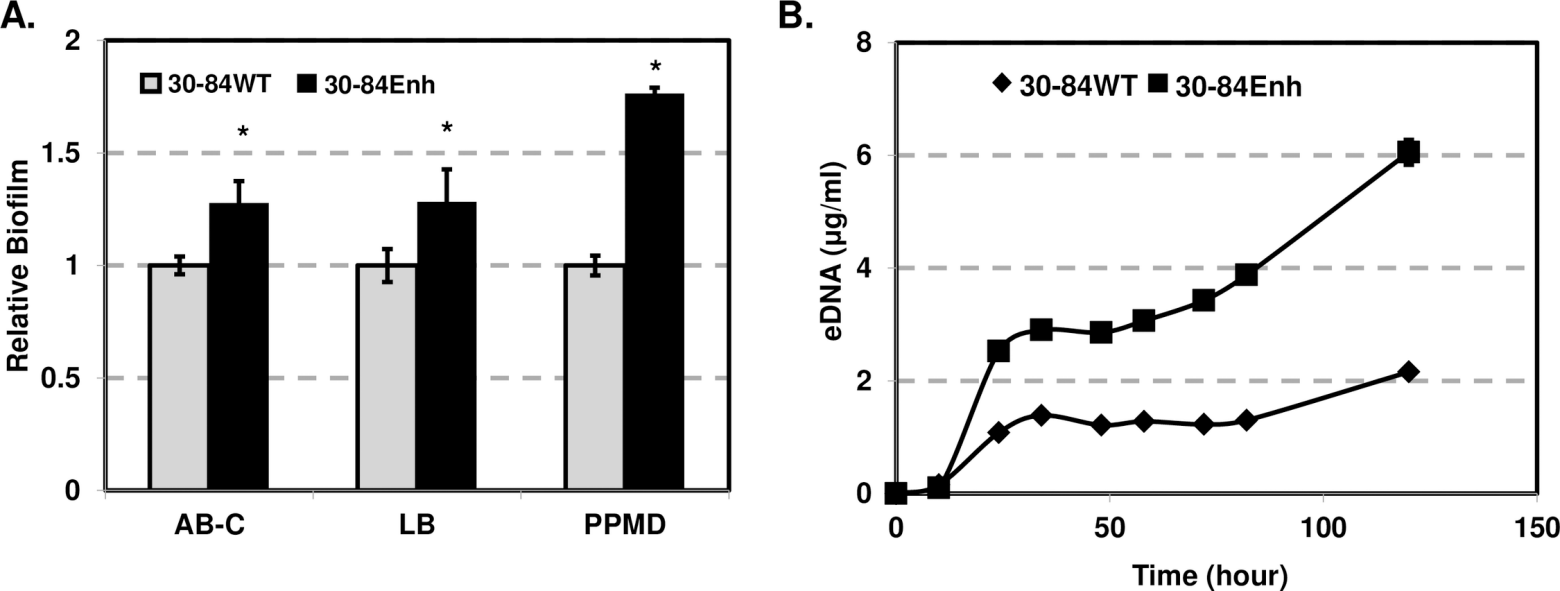
Effect of enhanced phenazine production on biofilm formation and eDNA release. **(A)** Biofilm formation by the wild-type (WT) and 30-84Enh. Bacteria were grown in AB-C, LB and PPMD in static plates for 48 h. Attached cells were stained with crystal violet and quantified by OD_540_. Relative biofilm was calculated by standardizing to the wild-type (assigned a value of 1). Data represent average of six replicates from two separate experiments with standard errors. Asterisks indicate significant differences as determined by unpaired t-test (P < 0.05). (**B)** Release of eDNA by the 30-84WT and 30-84Enh. Cultures were grown in AB minimal for 120 h with rapid agitation. Samples were taken periodically and the concentration of eDNA was quantified using a Quibit (Invitrogen) fluorometer. In some cases, error bars do not exceed the size of the symbol. These experiments were repeated with similar results.

Our previous research showed that altering the ratio of phenazines produced by *P*. *chlororaphis* 30–84 affected the attachment, density and structure of biofilms formed on solid surfaces as well as matrix production in floating biofilms [16, 17, 19]. To examine the effects of enhanced phenazine production on biofilm formation, the wild-type and 30-84Enh were grown in static culture. Strain 30-84Enh formed significantly more biofilm than the wild-type in all medium tested (Fig 5A). Since eDNA release is a key component of biofilm matrix [18, 19, 50], we compared eDNA release by the wild-type and 30-84Enh. Strain 30-84Enh produced significantly more eDNA than the wild-type over time (Fig 5B).

We also tested whether wheat root colonization by the 30-84Enh strain differed from the wild-type using two inoculation methods: root-dip (3-day old seedlings) and soil inoculation (inoculum mixed with soil). Bacterial populations of root-dip inoculated wild-type and 30-84Enh on roots were similar (9.60 ± 0.2 and 9.48 ± 0.1 log CFU/g of roots, respectively, n = 8, p>0.05) after 30 days of growth. Wheat roots also recruited similar populations of wild-type and 30-84Enh from the soil after 24 days (7.72 ± 0.12 and 7.89 ± 0.06 log CFU/g of roots, respectively, n = 10, p > 0.05). These results suggest that enhanced phenazine production did not alter *P*. *chlororaphis* 30–84 colonization or persistence on roots.

### Improved biological control ability to inhibit fungal growth and take-all suppression

As expected, enhanced phenazine production resulted in greater *in vitro* fungal inhibition of *Ggt*. Zones of fungal growth inhibition were significantly greater for 30-84Enh than the wild-type (8.2 **±** 0.4 mm and 6.5 **±** 0.3 mm, respectively, p<0.005, n = 9). To determine whether the increased phenazine production also results in enhanced disease suppression, the wild-type and 30-84Enh were seed inoculated and planted into a soil mix infested with *Ggt* as described previously [1, 51]. Strain 30-84Enh inoculated seedlings had significantly improved take-all suppression (reduced lesions on roots and disease symptoms on leaves) compared to the wild-type or the negative control (no bacterial inoculant) (Table 2). These results confirmed that enhanced phenazine production resulted in greater pathogen inhibition and disease suppression.

**Table 2:** Bacterial populations and take-all disease symptoms on wheat roots inoculated with *P*. *chlororaphis* 30–84 wild-type or 30-84Enh.

Treatment	Inoculum	Population	Take-all
	(CFU/seed)^a^	(CFU/g of root)^b^	Disease symptoms^c^
30-84WT	2.0 × 10^7^	3.7 × 10^8^	2.9 ± 0.1 B
30-84Enh	1.3 × 10^7^	3.3 × 10^8^	1.8 ± 0.2 C
Methyl cellulose	ND^e^	ND	4.7 ± 0.1 A

^a^ Mean of initial inoculum dose of each bacterial treatment on wheat seeds.

^b^ Mean of bacterial population of each strain on wheat roots at the time of the disease evaluation (20 days).

^c^ After 20 days of growth, roots were thoroughly washed and evaluated for disease severity on scale of 0 (no disease)– 5 (nearly dead). The values are average of two separate experiments (8 plants per each experiment) with standard errors.

Values with the different letters indicate significant differences as determined by a Tukey’s multiple comparison test (P < 0.05).

^e^ ND = Not detected.

## Discussion

In *P*. *chlororaphis* 30–84, phenazines are essential for rhizosphere colonization, biofilm formation, pathogen inhibition and disease suppression [1, 16, 17, 39]. Given the importance of the bacterial functions for which phenazine production is vital, much attention has focused on phenazine regulation, including the influence of quorum sensing and other phenazine regulatory systems (e.g. transcriptional/translational regulatory proteins or ncRNAs) [22, 23, 27, 35, 40, 42, 52]. Analysis of the sequence between the divergently oriented *phzR* and *phzX* promoters identified multiple repetitive sequences and other features within the 5’-UTR of *phzX* and *phzR* capable of producing secondary structures (S2A Fig), which may alter gene expression by one or both promoters. Transcriptional fusion of *phzX* derivatives with deletions were analysed for their effect on gene expression. Deletion of the 90-bp sequence containing the third and the fourth palindrome sequences (*Ssp*I repeat sites) and the second direct repeat sequence resulted in 4-fold greater expression by the transcriptional reporter compared to the reporter with the wild-type sequence (Fig 2). These results indicate that this region is involved in negatively modulating phenazine gene expression. However, modifications of specific sequence motifs did not alleviate reduced expression significantly (S1B Fig), likely because the AT-rich nature of this region created other secondary structures. This high level of overall secondary structure may explain why deletion of this entire region was necessary to alleviate reduced expression. Deletion of this region from the *P*. *chlororaphis* 30–84 chromosome resulted in the construction of strain 30-84Enh that produced significantly more phenazine than wild-type (Fig 3A and 3C). Importantly, phenazine production by 30-84Enh still required activation via quorum sensing and still required a functional AHL synthase. Deletion of the 90-bp sequence also significantly improved AHL production and *phzR* and *phzI* transcript abundances (Fig 3B and 3D), which probably contributes to the increased phenazine production by 30-84Enh. Our evidence suggests that deletion of this sequence facilitated higher expression of the phenazine biosynthetic operon as well as the divergently transcribed *phzR*, the latter leading to increasing PhzR levels and enhancing interaction with the AHL signal. Since the promoters of both *phzI* and *phzX* have *phz* boxes, this would lead to higher *phzI* and phenazine gene expression.

In many plant-beneficial *Pseudomonas* species, Gac/Rsm-mediated regulation plays a crucial role in the control of secondary metabolites [27, 28, 33, 35, 36, 38, 53, 54]. However, the mechanisms by which RsmA/E mediate regulation of phenazine biosynthesis are somewhat species specific and can be either positive or negative. For example, RsmA differentially regulates the expression of the two phenazine biosynthetic operons in *P*. *aeruginosa* M18 [35]. RsmA binds upstream of the RBS of the *P*. *aeruginosa* M18 *phz2* operon, resulting in enhancement of translation via destabilization of the stem loop structure. In contrast, RsmA binds motifs near the RBS of the *P*. *aeruginosa* M18 *phz1* operon and negatively regulates its expression, likely due to RsmA blocking the RBS and/or targeting mRNA degradation as observed in other studies [28, 29, 55–57]. Similar to RsmA regulation of the *P*. *aeruginosa* M18 *phz1* operon, translational fusion assays suggest that RsmE targets the stem-loop structure in the *P*. *chlororaphis* 30–84 5’-UTR of phenazine biosynthetic operon, and deletion of the 90-bp enables 30-84Enh to avoid RsmE-mediated translational repression. However, we observed that in an *rsmE* mutant the translational activity of the wild-type reporter was still less than that of the reporter with the 90-bp deletion, indicating that disruption of *rsmE* only partially relieves repression. This finding reaffirms that the 90-bp sequence is also involved in interactions with transcriptional regulatory mechanisms that contribute to the reduced expression of the phenazine biosynthetic operon. The specific point of interaction between RsmE/*rsmZ* and the 5’-UTR of phenazine biosynthetic operon is currently being investigated.

### Why do only 2OHPCA producers contain the conserved sequence that negatively modulates phenazine production?

One interesting finding of this study was that the multiple repetitive sequences we identified within the 5’-UTR of phenazine biosynthetic operon in *P*. *chlororaphis* 30–84 are also highly conserved among other 2OHPCA producing strains (e.g. having a single phenazine operon with *phzO* downstream of the *phz* operon), but are absent or differ from strains that produce other types of phenazines (Fig 1A and 1B). This led us to speculate why strains that produce 2OHPCA such as *P*. *chlororaphis* 30–84 and require quorum sensing for phenazine production modulate expression of the biosynthetic genes via the conserved presence of this upstream sequence. One hypothesis is that the cluster of phenazine regulatory (*phzI/phzR*) and biosynthetic genes (including *phzO*) share a common inheritance among 2OHPCA producers, with selection pressure for maintaining the conserved sequence. It was noted previously that despite the benefits of phenazine production to the ecological fitness of phenazine producers [1, 13, 16, 17, 39], phenazine production is stressful for the producing strain [45, 58]. It is interesting to speculate that some phenazine derivatives are more stressful to the producer than others. For example in *P*. *aeruginosa*, phenazine production, especially pyocyanin, promotes eDNA release presumably via the generation of reactive oxygen species (ROS) leading to cell lysis [59]. Wang et al. [19] demonstrated that cell lysis and release of eDNA by *P*. *chlororaphis* 30–84 was greater among derivatives that produced 2OHPCA compared to those that produced only PCA or do not produce phenazines. Additionally, cell lysis and eDNA release increased in derivatives producing more 2OHPCA. Consistent with the hypothesis that 2OHPCA production might be stressful for the cell, transcriptomic analysis revealed that as compared to derivatives that produce only PCA or do not produce phenazine, production of 2OHPCA by wild-type and the 2OHPCA overproducer significantly increased expression of genes involved in oxidative stress response and management, including ROS detoxifying enzymes, efflux pumps, DNA repair/modification enzymes, as well as a gene cluster encoding a bacteriophage-derived pyocin under the control of a stress-inducible promoter [19]. Similarly, 30-84Enh released up to 3-fold more eDNA than the wild-type (Fig 5B). Because production of 2OHPCA may be stressful in certain environments, 2OHPCA producers may have evolved or maintained the unique 5’-UTR sequence for tighter regulation of phenazine production as compared to strains that do not produce 2OHPCA. Interestingly, 30-84Enh is not altered in growth in shaking culture or in survival on wheat roots in growth chamber studies compared to the wild-type, suggesting that overproduction of phenazines under quorum sensing control is not detrimental in these environments.

### *P*. *chlororaphis* 30-84Enh improves biological control

Phenazines have been studied extensively for their broad spectrum antibiotic activity against a diversity of soil-borne plant pathogens. We hypothesized that increased phenazine production should result in enhanced biological control. Consistent with this hypothesis, 30-84Enh has greater ability to inhibit fungal growth of the *Ggt in vitro* as well as take-all disease suppression than the wild-type (Table 2). Encouragingly for biological control applications, creation of 30-84Enh via the deletion of 90-bp sequence resulted in a strain with improved ability to form biofilms, produce eDNA, inhibit target pathogens and suppress disease, without deficiencies in growth rate or the ability to colonize wheat roots under our test conditions. The latter may be due to control of phenazine production by quorum sensing regulation, which determines the timing of phenazine production. Thus rather than achieving enhanced phenazine production via constitutive expression of genes in the chromosome or via the insertion of foreign genes *in trans* [60, 61], deletion of the modulating sequence may be both ecologically germane and environmentally safer for a biological control agent. The benefits of this approach include reducing potential interference in the establishment of a beneficial rhizosphere microbiome via constant phenazine antibiotic production and minimizing the possibility for horizontal gene transfer [62]. Future studies will consider whether these capabilities result in better suppression of other plant diseases or more reliable interactions with host plants and their rhizosphere microbiomes, contributing to enhanced plant health.

## Materials and methods

### Bacterial strains, plasmids and growth conditions

Bacterial strains and plasmids used in this study are described in Table 1, and primers used in this study are listed in S1 Table. A spontaneous rifampicin-resistant strain of *P*. *chlororaphis* 30–84 and its derivatives were grown at 28°C in Luria-Bertani medium (LB) (5 g of NaCl per liter), AB minimal media (AB), AB amended with 2% casamino acids (AB-C) (Difco, Franklin Lakes, NJ), or pigment production medium-D (PPM-D) [16, 17, 40, 44]. Where applicable, antibiotics were used in the following concentrations: gentamicin (Gn; 50 μg/ml), kanamycin (Km; 50 μg/ml), rifampicin (Rif; 100 μg/ml), tetracycline (Tc; 50 μg/ml) and piperacillin (Pip; 50 μg/ml) for *P*. *chlororaphis* 30–84, and ampicillin Ap (100 μg/ml), Gn (15 μg/ml), Tc (25 μg/ml) for *E*. *coli*.

### DNA manipulations, sequence analysis, and PCR

Standard methods were utilized for plasmid DNA isolation, restriction enzyme digestion, ligation, transformation, and agarose gel electrophoresis [63]. Plasmids were introduced into *P*. *chlororaphis* 30–84 and its derivatives using either triparental matings or electroporation using methods described previously [1, 40]. Standard polymerase chain reaction (PCR) were carried out using FideliTaq DNA polymerase (Affymetrix, Santa Clara, CA) as described previously [45]. DNA sequencing was performed by the Laboratory for Genome Technology within Institute for Plant Genomics and Biotechnology, Texas A&M University using an ABI 3130xl Genetic Analyzer.

### Phylogenetic analysis

To obtain the nucleotide sequence upstream of phenazine biosynthetic operon, the 250 bp nucleotide sequence upstream of the translation start site of *phzX* were blasted against the Pseudomonas Genome Database (www.pseudomonas.com) and National Center for Biotechnology Information (NCBI, https://www.ncbi.nlm.nih.gov/). A total of 27 phenazine producing strains were retrieved and analyzed for the types of a phenazine modifying enzyme. The nucleotide sequences (250 bp flanking sequences from the translation start site of each strain’s phenazine biosynthetic operon) were aligned and edited using MUSCLE (MEGA7). MEGA7 was used to build a, Maximum Likelihood (ML) trees based on the Tamura-Nei Model [64] [65]. ML bootstrapping was performed with 1,000 replicates to assess the relative stability of the branches.

### Construction of *phzX* transcriptional fusion derivatives

To determine the function of the 5’-UTR of phenazine biosynthetic operon in phenazine gene expression, a 1.5-Kb DNA fragment contains a deletion of 90-bp in the 5’-UTR of phenazine biosynthetic operon was synthesized (GeneScript, Piscataway, NJ) and obtained as pUC57-Enh (Table 1). This fragment lacks 90-bp of sequence (including both *Ssp*I sites and the second direct repeat) starting at the +8 bp (from the TSS) to the RBS of *phzX*, but maintaining the endogenous RBS and start codon of PhzX. PCR fragments of 271 bp and 181 bp containing sequence from -124 to +147 of *phzX* were amplified from genomic DNA of *P*. *chlororaphis* 30–84 and pUC57-Enh using the primers, phzXF1/phzXR1 (S1 Table) and cloned into the promoter trap vector pGT2-*lacZ* resulting plasmids pJMYX1 and pJMYX2, respectively (Fig 2 and Table 1). To determine the function of upstream sequence of phenazine biosynthetic promoter region, PCR fragments of 354bp (from -124 to +230), 310bp (from -80 to +230), and 274bp (from -44 to +230) containing the promoter of phenazine biosynthetic operon, 5’-UTR and 115bp of *phzX* coding sequence were amplified from *P*. *chlororaphis* 30–84 genomic DNA using the following primer sets, phzXF1/phzXR2, phzXF2/phzXR2 and phzXF3/phzXR2, respectively (S1 Table) These fragments were cloned into the promoter trap vectors pKT2-*lacZ*, creating the plasmids pJMYX3, pJMYX4 and pJMYX5, respectively (Table 1 and Fig 2). The plasmids pKT2-*lacZ* and pGT2-*lacZ* contain a promoterless *lacZ* gene with its own ribosome binding site (RBS) located downstream of multiple cloning locus, which enables the study of transcriptional activities.

All transcriptional fusion plasmids were separately introduced into 30-84Ice via triparental mating and the transcriptional activity was determined by β-galactosidase activity [66] in 30-84Ice after 24 h growth in LB with rapid agitation. Strain 30-84Ice was used for the transcriptional fusion assays because it contains a *phzB*::*inaZ* insertion (Table 1) and as a consequence does not produce phenazine, which interferes with the β-galactosidase assay.

### Generation of a phenazine enhanced mutant

In order to generate the phenazine enhanced mutant strain, the 1.5 Kb sequence from pUC57-Enh was cloned into the vector, pLAFR3, generating pLAF-phzEnh (Table 1). This 1.5 Kb fragment contains flanking regions upstream from the *Eco*RV site in the first half of *phzR* and downstream to the *Bam*HI site at the 3’-end of *phzY* (the second gene of *phz* operon) to facilitate homologous recombination. The pLAF-phzEnh plasmid was introduced into *P*. *chlororaphis* 30–84 strains containing pUCP18-RedS via triparental mating, and the chromosomal 90-bp deletion of 5’-UTR of phenazine biosynthetic operon was obtained with the support of λ phage recombinases [40, 48]. A dark orange colony (for 30-84Enh) from the PPMD plate or a dark blue colony (for 30-84ZN-Enh) from PPMD plate supplemented with 2% X-gal were chosen, and pUCP18-RedS plasmid (containing *sacB*) was cured by counter selection on LB plates supplemented with 5% sucrose. A Tc^s^, Pip^s^, and Sucrose^R^ colony was chosen and mutation was verified by PCR and sequencing.

### Construction of an *rsmE* deletion mutant

To inactivate *rsmE* gene in *P*. *chlororaphis* 30–84, fragments of upstream (611 bp) and downstream (706 bp) of the *rsmE* open reading frame (ORF) were amplified from *P*. *chlororaphis* 30–84 genomic DNA using primers RsmEUPF/RsmEUPR and RsmEDWF/RsmEDWR (S1 Table). These fragments were designed to carry a *Kpn*I site that permitted the insertion of a kanamycin resistance cassette at the 3’ end of upstream fragment and 5’ end of downstream fragment with 183 bp deletion of *rsmE* ORF. Each fragment was simultaneously cloned into the pEX18Ap (Table 1). A 961 bp fragment containing kanamycin resistance cassette was PCR amplified from pUC4K (Table 1) using the primers, KmKpnF/KmKpnR, and inserted into *Kpn*I site between upstream and downstream fragments in pEX18Ap. The resulting plasmid, pEX18Ap-rsmEKO (Table 1), was electroporated into *P*. *chlororaphis* 30–84, and mutant was selected for by amending LB plates with the appropriate antibiotics. A Km^R^, Pip^s^, and Sucrose^R^ colony was chosen and the *rsmE* mutation was verified by PCR and sequencing.

### Construction of translational fusion vector with 90-bp deletion of the 5’-UTR of phenazine biosynthetic operon

To determine the function of the 5’-UTR on translation of the phenazine biosynthesis genes, translation fusion vectors were created containing the sequence from the phenazine biosynthetic operon promoter to the 20^th^ codon of PhzX with or without 90-bp sequence of 5’-UTR of phenazine biosynthetic operon (Fig 4B). The fragments with the 90-bp sequence (299 bp) and without the 90-bp sequence (209 bp) were PCR amplified from genomic DNA of *P*. *chlororaphis* 30–84 and pUC57-Enh, respectively, using primer set phzXF1/phzXR3 (S1 Table). The products were cloned, in frame, with the 8^th^ codon of *lacZ* in the translational fusion vector, pME6015 [34, 45], resulting pJMYX7 and pJMYX8 respectively (Fig 4B and Table 1). These plasmids were introduced in the wild-type, 30-84W and 30-84RsmE via electroporation, and translational activities were determined by β-galactosidase activity.

### RNA preparation for quantitative PCR

To isolate RNA from the wild-type and 30-84Enh, single colonies of each strain were grown with rapid agitation at 28°C in 3 ml of AB-C broth. When cell density reached OD_620_ = 1.8, 1 ml aliquots of each sample were mixed with 2 ml of Qiagen RNA Protect reagent (Qiagen, Hilden, Germany) to stabilize bacterial RNA, and cells were harvested by centrifugation for 10 min at 2400 x g. Total RNA was extracted using a Qiagen RNeasy Mini Kit (Qiagen) according to the manufacturer’s recommended protocol. The genomic DNA was removed using on-column DNase-I digestion (Qiagen). Five micrograms of total RNA were reverse-transcribed using random primers (Invitrogen Life Technologies, Carlsbad, CA) and Superscript III (Invitrogen) at 50°C for 1 h and inactivated at 75°C for 15 min. For the negative control, the same reaction was performed using sterilized water instead of reverse transcriptase.

### qPCR methods and analysis

SYBR Green reactions were performed using the ABI 7900 HT Fast System (Applied Biosystems, Foster City, CA) in 384 well optical reaction plates. Quantitative PCR (qPCR) assays were performed to measure the expression levels of the target genes as previously described [45]. Briefly, aliquots (1 μl) of cDNA (2 ng/reaction) or negative controls were used as template for qPCR reactions with Fast SYBR Green PCR Master Mix (Applied Biosystems) and primers (500 nM final concentration). qPCR amplifications were carried out at 50°C for 2 min, 95°C for 10 min, followed by 40 cycles of 95°C for 15 sec and 60°C for 1 min, and a final dissociation curve analysis step from 65°C to 95°C. Technical replicate experiments were performed for each of the biological samples, in triplicate. Amplification specificity for each reaction was confirmed by the dissociation curve analysis. The Ct values were used for further ΔΔCt analysis. The 16S rDNA was used as a reference gene to normalize samples. A relative quantification value was calculated for each gene with the control group as a reference [27, 40, 45].

### Quantification of phenazine and AHL production

*P*. *chlororaphis* 30–84 strains were grown at 28°C in AB minimal, LB and PPMD broth with rapid agitation. Phenazines were extracted from cell-free supernatants as described previously [1, 16, 45]. Phenazines concentration was calculated via serial dilution of the extract at absorbance of 367 nm. Total AHLs were extracted as described previously [39, 45] from 5 ml cultures, which were grown at 28°C with shaking in AB minimal, LB and PPMD broth. AHL production was quantified by inoculating the extracted AHLs with the AHL-specific reporter strain 30-84I/Z (*phzI*^*-*^, *phzB*::*lacZ*). 30-84I/Z is deficient in AHL production due to mutation of AHL synthase gene *phzI*, but responds to AHL by expressing the reporter gene *lacZ*. β-galactosidase activity was determined on cultures grown at 28°C for 24 h with rapid agitation.

### Construction of *phzR* transcriptional reporters

In order to determine whether the 90-bp of the 5’-UTR of phenazine biosynthetic operon also negatively influence *phzR* expression, *phzR* transcriptional fusions were constructed. PCR fragments containing a sequence from -359 bp to +155 of *phzR* was amplified from genomic DNA of the wild-type and 30-84Enh using the primers, phzRF-phzRR (S1 Table). The 514 bp and 424 bp PCR amplicons containing *phzR* promoter region with or without the 90-bp of the 5’-UTR of *phzX*, respectively, were ligated into promoter trap vector pGT2-*lacZ* to make the *phzR* transcriptional fusion vectors, pJMYR1 and pJMYR2 (Table 1 and S4 Fig). These reporters were separately introduced into 30-84Ice by triparental mating, and transcriptional activities were determined by β-galactosidase activity.

### Microtiter plate biofilm assay and eDNA quantification

To measure the ability of strains 30–84 wild-type and 30-84Enh to form a biofilm, static biofilm assays were conducted in 24-well polystyrene microtiter plates. Briefly, overnight cultures grown in 3 different media (AB-C, LB and PPMD) were adjusted to an OD_620_ of 0.8 with fresh medium. The adjusted cultures were diluted 1:100 into the appropriate media, and 1.5 ml of the dilution inoculate was transferred into 24-well plates. Plates were incubated at 28°C without shaking. After 48 h, the adherent cell population was quantified by crystal violet staining as described previously [17, 67].

The concentration of eDNA was determined quantitatively using Qubit 2.0 Fulorometer (Invitrogen), as described previously with few modification [19]. Briefly, overnight cultures grown in AB-C broth at 28°C with agitation were adjusted to an OD_620_ of 0.8. The adjusted cultures were re-inoculated at a 1:100 dilution into 20 ml AB-C broth. Cultures were grown at 28°C with rapid agitation and sampled every 8–12 h. Cell-free supernatant by centrifugation and filtration were mixed with double-strand DNA fluorescent dyes (dsDNA BR) from Qubit (Invitrogen Life Technologies, Carlsbad, CA), and the concentration of eDNA was quantified using Qubit 2.0 Fluorometer (Invitrogen Life Technologies). The amount of eDNA was reported as μg/ml.

### Root colonization assay

To determine the ability of colonizing to the host plant, the wild-type and 30-84Enh were inoculated to wheat (cultivar TAM112) by two different methods. For the root-dip inoculation methods, bacterial cultures were grown in 10 ml KMB broth for 24 h, and inoculum was standardized to OD_620_ = 0.8 (ca. 2 x 10^9^) in sterile 1X PBS. Seeds were surface sterilized as previously described [44], and surface sterilized seeds were pregerminated on sterilized germination paper for two days. Seedling roots were dipped into the bacterial solution for 10 min, and sown into 25 × 200 mm cone-tainers that contain a natural wheat rhizosphere (Uvalde, TX) soil mix (soil: sand, 2:1, v:v). Plants were grown for 30 days before the entire root system was processed for the CFU calculation, as described previously [44]. For the soil inoculation method, bacterial cultures were prepared as described above. Bacteria cultures were washed 3 times with sterilized water, and inoculum were thoroughly mixed with natural wheat rhizosphere soil mix (soil: sand, 2:1, v:v) for final bacterial population of 10^6^ CFU/g of soil. Pregerminated wheat seedlings (two days) were sown into the bacterial amended soil mix and were grown for 24 days. Bacterial populations were collected from the entire root system and quantified by CFU. Total populations were determined by serial dilution on LB agar amended with rifampicin.

### Fungal inhibition and take-all suppression assay

To quantify the ability of strains 30–84 wild-type and 30-84Enh to inhibit the take-all causal agent *Ggt* strain ARS-A1, an *in vitro* dual culture assay was conducted as described previously [45]. After 7 days of co-culture on potato dextrose agar plates, the zone of inhibition was measured as the distance between edge of the bacterial colony and the fungal mycelium.

Assays to determine the ability of strains 30–84 wild-type and 30-84Enh to suppress take-all disease on wheat seedlings were conducted as described previously [1, 51]. Briefly, bacteria-coated seeds or control seeds (coated with methyl cellulose) were sown in tubes (25 × 200 mm) filled with 5g of sterilized vermiculate layer overlaid with 20 g of a natural wheat rhizosphere soil mix (soil: sand, 2:1, v:v) amended with *Ggt* colonized oat kernels fragments (0.85%, w/w). For the control, sterilized oat kernels were ground and amended to the soil mix with same amount (0.85%, w/w). Seeds (cv. TAM112) were covered with 1cm of sterilized vermiculate and incubated for 3 days at room temperature to facilitate germination. Seedlings were arranged in a complete randomized block design, and transferred to a growth chamber (16°C, 12 h dark-light cycle). After 20 days, root disease was evaluated on a scale of 0–5, where 0 = no disease and 5 = nearly dead. as described previously [1].

### Statistical analysis

All data presented are mean ± the standard error of the mean (STE) from at least two experiments. Data were analyzed using ANOVA and Fisher’s protected Least Significant Difference (LSD) test (P<0.05) or unpaired t-test. Data were processed with GraphPad Prism (GraphPad Software, San Diego, CA).

## Supporting information

S1 Table Oligonucleotides used for gene cloning and qPCR. (PDF)

S1 Fig Analysis of *phzX* expression in *P*. *chlororaphis* 30–84 using transcriptional fusion to the *lacZ* reporter gene. The region of the *P*. *chlororaphis* 30–84 chromosome that contains the promoter of the phenazine biosynthetic operon (*phzXYFABCD*) includes four palindromic sequences (solid arrowheads) and two direct repeats (open arrowheads). For each plasmid derivative, the rectangles with the diagonal lines indicate the sequence included in each derivative, the solid black rectangle represents the *lacZ* reporter gene sequence, and the black triangles under the *lacZ* reporter represent the ribosome binding site of *lacZ*. The transcriptional expressions of the *phz* promoter derivatives in 30-84Ice were determined via the β-galactosidase activities and presented as Miller Units. Data represent the average of eight replicates with standard errors. The different letters indicate significant differences by Fisher’s protected Least Significantly Difference (LSD) test (P < 0.05). **Note:** Expression of control plasmids pJMYX1 (Fig 2) and pJMYX3 were not significantly different.
(PDF)

S2 Fig Analysis of secondary structure prediction and the activity of promoter derivatives. **(A)** Potential secondary structure predicted from the RNA sequence spanning the transcription start site (+1) site to the *Sal*I site of *phzX*. The two *Ssp*I sites are marked with blue highlight, the second direct repeats are marked with red highlight, and the putative *phzX* RBS and start codon are marked with green highlight. **(B)** The pJMYX4 and its derivatives with specific sequence alterations fused to *lacZ*. The black solid arrows represent the two direct repeats (5’-CACCCCCAA-3’), DR1 and DR2. The black rectangles represent the RBS of *phzX*. The hollow solid arrows represent modified sequence motifs. The orange rectangles represent partial ORF of *phzX*. The hatched rectangles represent *lacZ* and its RBS (triangle); dotted lines indicate restriction enzyme sites. **(C)** The β-galactosidase activity of pJMYX4 promoter (control) and each derivative in 30-84Ice. Promoter activity is expressed in Miller Units as the average of 8 replicates with standard error. Values with the same letter do not differ significantly as determined by a Fishers protected Least Significantly Difference (LSD) test (P ≥ 0.05).
(PDF)

S3 Fig Characterizing phenazine production by 30-84Enh and 30-84ZN-Enh. **(A)** Phenazine production by *P*. *chlororaphis* 30–84 wild-type and 30-84Enh after 48 h on PPMD agar plates. **(B)** Overnight culture of 30-84ZN and 30-84ZN-Enh on LB broth supplemented with 2% X-Gal. **(C)** Expression of the *phz* biosynthetic operon in 30-84ZN and 30-84ZN-Enh. Expression of *phz* operon was quantified by β*-*galactosidase assay. Each bar represent means ± SE of six replicates from two independent experiments.
(PDF)

S4 Fig Map of the promoter sequences used to construct the PhzR transcriptional fusion reporters. Construction map for transcriptional fusion reporter plasmids, pJMYR1(control) and pJMYR2 (90bp deletion). These reporters containing flanking sequence from -359 to +155 including *phzR* promoter (-35 and -10), ribosome binding site, transcription and translation start site and the first 84 bp of the *phzR* gene. The rectangles with the diagonal lines indicate the sequence included in each derivative, the hollow rectangles indicate the 90-bp region not included in pJMYR2, the solid grey rectangle represents partial ORF of *phzR*, the solid black rectangle represents the *lacZ* reporter gene sequence, and the black triangles under the *lacZ* reporter represent the ribosome binding site of *lacZ*. Divergently transcribed *phzX* promoter and the relative nucleotide based on +1 of *phzX* are presented as grey.
(PDF)
