# Supplementary material for: An upstream sequence modulates phenazine production at the level of transcription and translation in the biological control strain Pseudomonas chlororaphis 30-84
Source: PLoS One. 2018 Feb 16;13(2):e0193063. doi: 10.1371/journal.pone.0193063 (PMC5815613; doi:10.1371/journal.pone.0193063)
Supplement: S1 Table — (PDF) [file pone.0193063.s001.pdf]

S1 Table. Oligonucleotides used for gene cloning and qPCR

Primers	Sequence (5'-3')
phzXF1	CGCGAATTCGAACCCGCCTCTTCACAATA
phzXF2	AATGAATTCGCAGCCAGCCCGCTCAT
phzXF3	TCACTGAATTCTCTGGTAGTTCCAC
phzXR1	CGCGGATTCATTAAAGCCGCTAGGGGAAA
phzXR2	AACAGGATCCGTCGACGCAGGCGATCTTT
phzXR3	GACCGTGGATCCATTTTTCTGGCGAAGTTC
phzRF	GAAGCGAATTCGGGCAGCATCCTCCTT
phzRR	CCTCAAGGATCCAGCCGTAAACTCCTGC
RsmEUPF	CGACGGAATTCTCGATCACTTGCGGCTCGATATTGCGC
RsmEUPR	AAGGAGAAGATCATGCTGGGTACCCCTTGA
RsmEDWF	ATGCTGGGTACCCCTTGAGCGTCATGAGCA
RsmEDWR	TGCTCATGACGCTCAAGGGGATCCCAGCATGATCTTCTC
KmKpnF	CGCGCGCGGTACCTGTGTCTCAAAATC
KmKpnR	CGCGCGCGGTACCTTTAGAAAAACTCATCG
16s RT1	ACGTCCTACGGGAGAAAGC
16s RT2	CGTGTCTCAGTTCCAGTGTGA
phzX RT2	AACCACTTCTGGGTGGAAAG
phzX RT2	ATCTTGCCGTCATCCAGTTC
phzB RT1	CTACATGGTGGTGGATGAAGAG
phzB RT2	GGTCTTGCCTTCGATGAAGTA
phzO RT1	CAGAGAGAAGAACCTGCTGATG
phzO RT2	GTGCGTGCTGTTCTTCAAAC
phzR RT1	CGCAAGGATAATCCCATCAG
phzR RT2	CACATTCCCTACCGCTGAAC
phzI RT1	CTACCTCCTGGCGTTCAATG
phzI RT2	GAAGCGAGTCATTTCCCAGA

[a] Underlined nucleotides are restriction sites added
