# Supplementary figures and images for: An upstream sequence modulates phenazine production at the level of transcription and translation in the biological control strain Pseudomonas chlororaphis 30-84
Source: PLoS One. 2018 Feb 16;13(2):e0193063. doi: 10.1371/journal.pone.0193063 (PMC5815613; doi:10.1371/journal.pone.0193063)

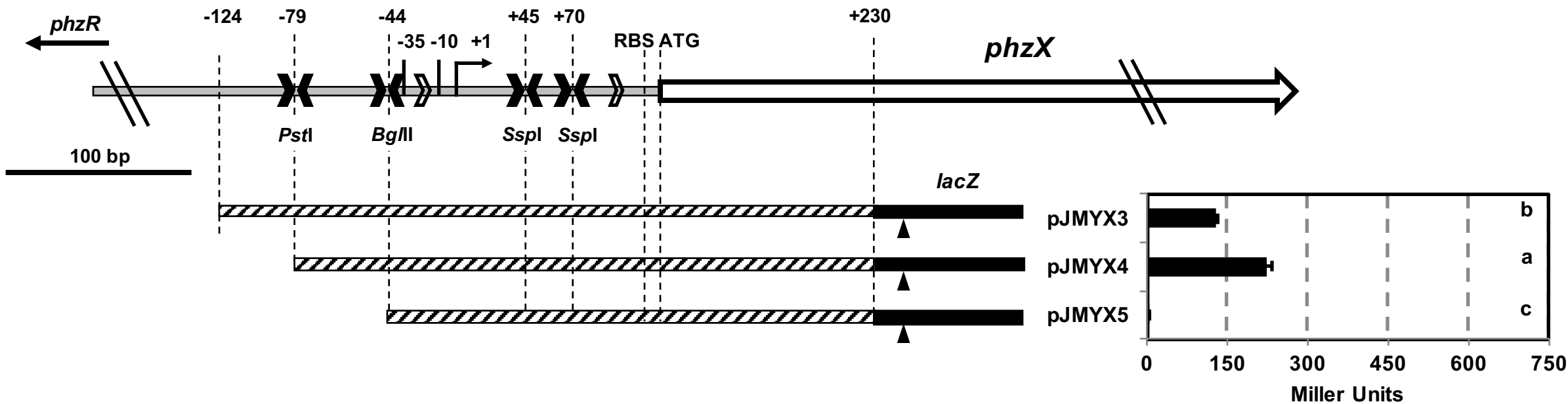

Supplement: S1 Fig — The region of the P. chlororaphis 30–84 chromosome that contains the promoter of the phenazine biosynthetic operon (phzXYFABCD) includes four palindromic sequences (solid arrowheads) and two direct repeats (open arrowheads). For each plasmid derivative, the rectangles with the diagonal lines indicate the sequence included in each derivative, the solid black rectangle represents the lacZ reporter gene sequence, and the black triangles under the lacZ reporter represent the ribosome binding site of lacZ. The transcriptional expressions of the phz promoter derivatives in 30-84Ice were determined via the β-galactosidase activities and presented as Miller Units. Data represent the average of eight replicates with standard errors. The different letters indicate significant differences by Fisher’s protected Least Significantly Difference (LSD) test (P < 0.05). Note: Expression of control plasmids pJMYX1 (Fig 2) and pJMYX3 were not significantly different. (PDF) [file pone.0193063.s002.pdf]

A.

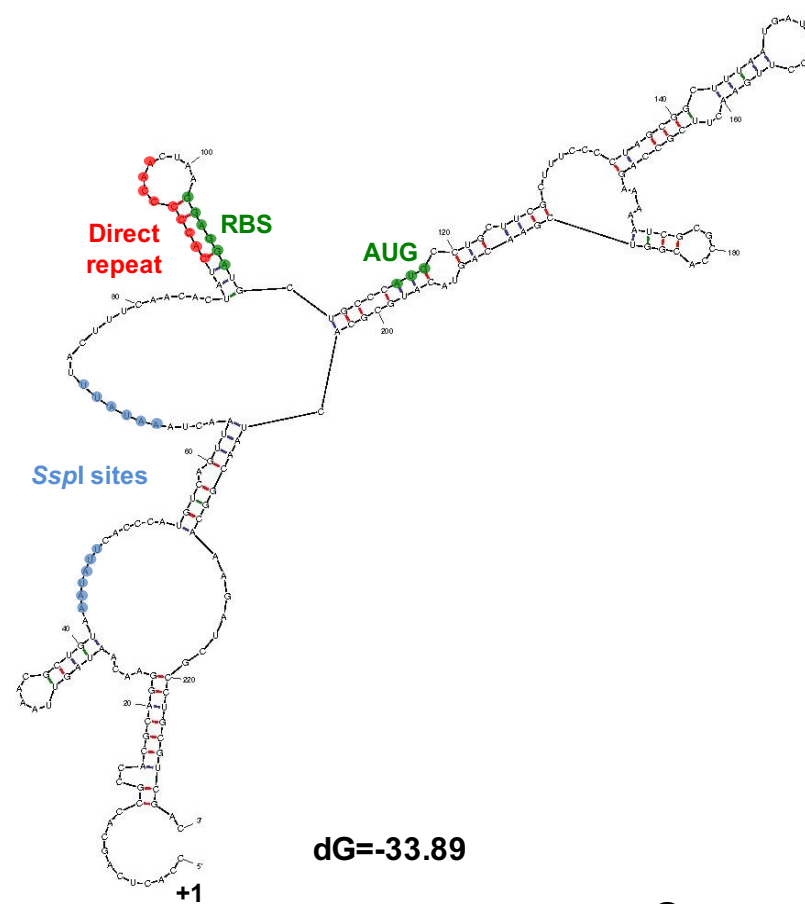

B.

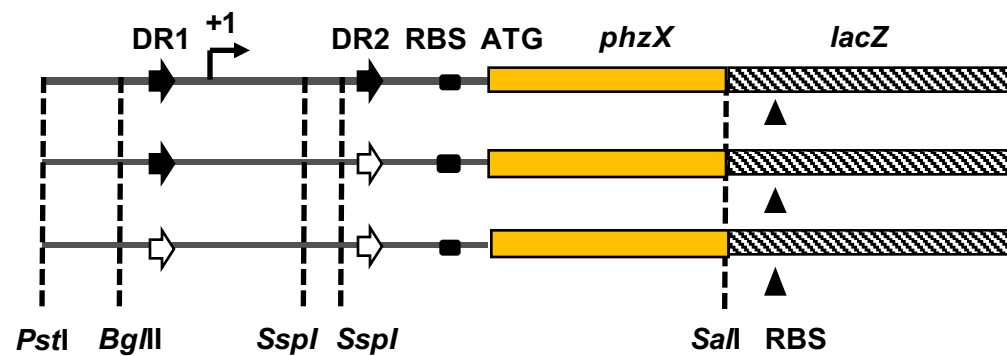

C.

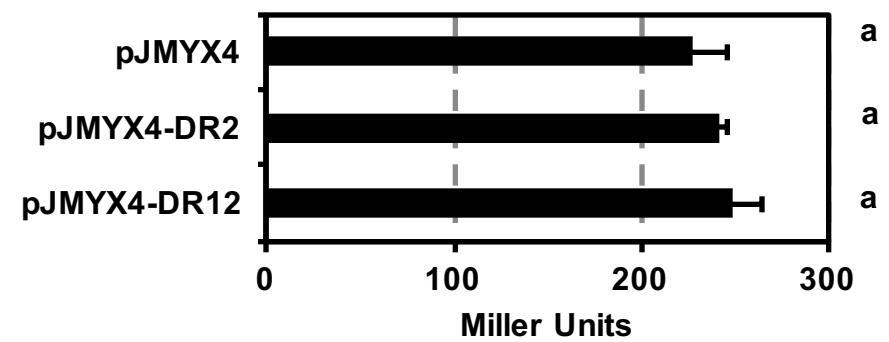

Supplement: S2 Fig — (A) Potential secondary structure predicted from the RNA sequence spanning the transcription start site (+1) site to the SalI site of phzX. The two SspI sites are marked with blue highlight, the second direct repeats are marked with red highlight, and the putative phzX RBS and start codon are marked with green highlight. (B) The pJMYX4 and its derivatives with specific sequence alterations fused to lacZ. The black solid arrows represent the two direct repeats (5’-CACCCCCAA-3’), DR1 and DR2. The black rectangles represent the RBS of phzX. The hollow solid arrows represent modified sequence motifs. The orange rectangles represent partial ORF of phzX. The hatched rectangles represent lacZ and its RBS (triangle); dotted lines indicate restriction enzyme sites. (C) The β-galactosidase activity of pJMYX4 promoter (control) and each derivative in 30-84Ice. Promoter activity is expressed in Miller Units as the average of 8 replicates with standard error. Values with the same letter do not differ significantly as determined by a Fishers protected Least Significantly Difference (LSD) test (P ≥ 0.05). (PDF) [file pone.0193063.s003.pdf]

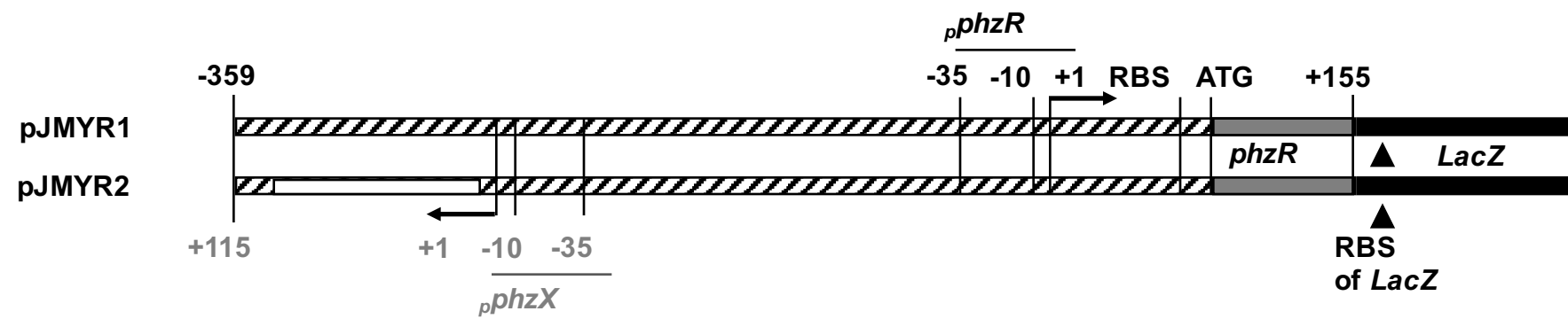

Supplement: S4 Fig — Construction map for transcriptional fusion reporter plasmids, pJMYR1(control) and pJMYR2 (90bp deletion). These reporters containing flanking sequence from -359 to +155 including phzR promoter (-35 and -10), ribosome binding site, transcription and translation start site and the first 84 bp of the phzR gene. The rectangles with the diagonal lines indicate the sequence included in each derivative, the hollow rectangles indicate the 90-bp region not included in pJMYR2, the solid grey rectangle represents partial ORF of phzR, the solid black rectangle represents the lacZ reporter gene sequence, and the black triangles under the lacZ reporter represent the ribosome binding site of lacZ. Divergently transcribed phzX promoter and the relative nucleotide based on +1 of phzX are presented as grey. (PDF) [file pone.0193063.s005.pdf]
