# Supplementary material for: An upstream sequence modulates phenazine production at the level of transcription and translation in the biological control strain Pseudomonas chlororaphis 30-84
Source: PLoS One. 2018 Feb 16;13(2):e0193063. doi: 10.1371/journal.pone.0193063 (PMC5815613; doi:10.1371/journal.pone.0193063)
Supplement: S3 Fig — (A) Phenazine production by P. chlororaphis 30–84 wild-type and 30-84Enh after 48 h on PPMD agar plates. (B) Overnight culture of 30-84ZN and 30-84ZN-Enh on LB broth supplemented with 2% X-Gal. (C) Expression of the phz biosynthetic operon in 30-84ZN and 30-84ZN-Enh. Expression of phz operon was quantified by β-galactosidase assay. Each bar represent means ± SE of six replicates from two independent experiments. (PDF) [file pone.0193063.s004.pdf]

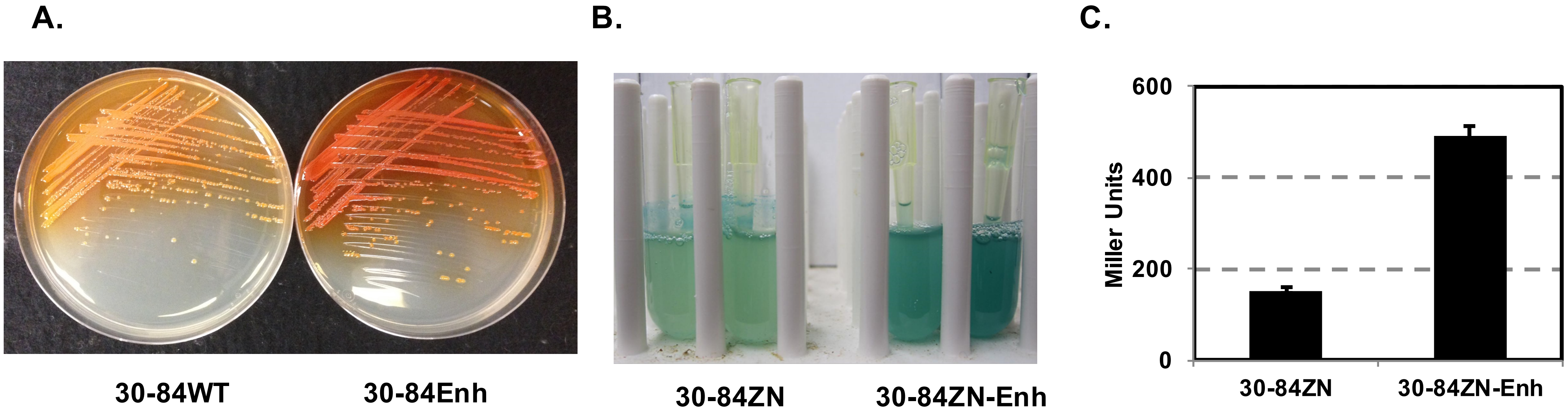

S3. Fig. Characterizing phenazine enhanced strains of 30-84Enh and 30-84ZN-Enh. A. Phenazine production by *P. chlororaphis* 30-84 wild type and 30-84Enh after 48 h on PPMD agar plates. B. Overnight culture of 30-84ZN and 30-84ZN-Enh on LB broth supplemented with 2% X-Gal. C. Expression of the *phz* biosynthetic operon in 30-84ZN and 30-84ZN-Enh. Expression of *phz* operon was quantified by β-galactosidase assay. Each bar represent means ± SE of six replicates from two independent experiments.
